# Head-Mounted Display-Based Application for Cognitive Training

**DOI:** 10.3390/s20226552

**Published:** 2020-11-17

**Authors:** José Varela-Aldás, Guillermo Palacios-Navarro, Rebecca Amariglio, Iván García-Magariño

**Affiliations:** 1 Department of Electronic Engineering and Communications, University of Zaragoza, 44003 Teruel, Spain; josevarela@uti.edu.ec; 2 Facultad de Ingeniería y Tecnologías de la Información y la Comunicación, Universidad Tecnológica Indoamérica, Ambato 180103, Ecuador; 3 Medical School, Harvard University, Boston, MA 02115, USA; ramariglio@mgh.harvard.edu; 4 Massachusetts General Hospital, Boston, MA 02114, USA; 5 Department of Software Engineering and Artificial Intelligence, Complutense University of Madrid, 28040 Madrid, Spain; igarciam@ucm.es

**Keywords:** cognitive rehabilitation, head-mounted display, immersive virtual reality, memory assessment, Oculus Go, serious game, wearable system

## Abstract

Virtual Reality (VR) has had significant advances in rehabilitation, due to the gamification of cognitive activities that facilitate treatment. On the other hand, Immersive Virtual Reality (IVR) produces outstanding results due to the interactive features with the user. This work introduces a VR application for memory rehabilitation by walking through a maze and using the Oculus Go head-mounted display (HMD) technology. The mechanics of the game require memorizing geometric shapes while the player progresses in two modes, autonomous or manual, with two levels of difficulty depending on the number of elements to remember. The application is developed in the Unity 3D video game engine considering the optimization of computational resources to improve the performance in the processing and maintaining adequate benefits for the user, while the generated data is stored and sent to a remote server. The maze task was assessed with 29 subjects in a controlled environment. The obtained results show a significant correlation between participants’ response accuracy in both the maze task and a face–pair test. Thus, the proposed task is able to perform memory assessments.

## 1. Introduction

Reality refers to the physical world in which the human being develops and lives with other elements, using all his senses. Although the activities carried out by a user still require their presence, technological means of communication have been designed that have changed the way to interact with the environment [1,2]. With the advancement of science, extended reality (XR) systems have been developed that allow reproducing environments, people, and objects with high fidelity using a computer [3,4]. This term includes virtual reality (VR), augmented reality (AR), and mixed reality (MR) [5]. VR refers to complete three-dimensional virtual representations of the real world or objects within it [6]. The first applications focused on entertainment, although they explored other areas such as marketing and engineering [7,8,9]. Continuing with the evolution and in order to provide the user with a range of experiences that resemble real ones, immersive environments have been implemented [4]. This allows the user to have the perspective of living in one reality and experiencing another synthetically at the same time.

Approaches based on interaction with a user, body language, and gestures have allowed objects to be manipulated in VR. A full immersive system can incorporate a wide field of view, high resolution, a head-mounted display (HMD), and auditory and tactile or force feedback [10]. Unlike a real scenario, the applications that can be performed with a virtual environment are unlimited. Understanding a concept, learning a procedure or doing a recreational activity are possible thanks to VR. Its application is multidisciplinary and in areas such as education, it allows the development of practices in medicine, biology, military, geology, aeronautics, engineering, and so forth [6]. In Chen et al. [11], Parong et al. [12], and Park et al. [13], motivational, attitudinal and behavioral changes can be seen in the student–teacher interaction in the learning process. Virtually exploring scenes that in reality would not be possible, stimulates the student to better understand and memorize the academic content [9,14].

The benefits that have been obtained in the field of medicine, contribute to preventing and maintaining the state of health of the person [15]. In diseases like Parkinson’s [16,17,18], challenging activities are performed in a safe environment, as a tool for neurorehabilitation of the upper and lower extremities. As a complement, qualitative data can be obtained on the emotions and experiences that patients feel during experiments [19]. The utility of immersive virtual reality (IVR) systems in the rehabilitation of traumatic brain injuries can be seen in the review presented by Aida et al. [20]. People who have suffered a stroke are also subject to rehabilitation processes, replacing conventional practices with systems that simulate the development of games or interactive activities. [21,22,23,24,25]. In psychology, social signals can be analyzed in a virtual world that requires contrast with the real one [26]. It must be taken into account that the content developed must be personalized and optimized to stimulate the individual in the desired way [27].

Despite the advantages offered by IVR systems, the underlying methodologies and theories used to influence users must be understood, since the use of immersive technology in the long term could affect their behavior, perception, and motor skills [28,29]. These systems can be used to avoid bad habits, addictions or aggressive behavioral responses [30,31,32]. Cognitive stimulation is a fundamental strategy for the improvement and rehabilitation of mental capacities and executive functions, such as memory, attention, processing speed, and contextual learning [33,34], evaluating in this way the behavior of the participants and improving the conditions that unbalance their state of mental well-being [35]. Although the literature does not allow establishing the full effectiveness of IVR to counteract disorders such as dementia, potential distractors are reduced and the experience of people with mild cognitive impairment is improved [36].

IVR is considered a valid alternative tool for the rehabilitation of cognitive decline at all ages. De Luca et al. [37] described the case of a 15-year-old adolescent with traumatic brain injury (TBI) treated using CAREN (Computer Assisted Rehabilitation Environment), who obtained a significant improvement in his specific cognitive and motor domains. Similarly, in De Luca et al. [38], outstanding results in semi-immersive virtual rehabilitation in young adults (averaging 40 years old) who suffered from TBI are discussed. When performing cognitive therapies in older adults, positive emotional changes have been observed, with an opening to a broader application, as described by Appel et al. [26]. Although the immersive experience can produce side effects (dizziness, nausea, headache, and stomachache), it is more likely that non-immersive sessions are carried out in them, as presented by Gamito et al. [39] and Liu et al. [40]. The effect obtained can be influenced by age, therefore, Shakare et al. [41] develop a 3D environment for navigation and cycling, tested on participants of various ages (two groups of 26 and 64 years on average) to assess their experiences, as a precedent for future work.

Memory is evaluated using neuropsychological exams; VR offers tools to apply evaluations with greater cognitive demands of the participant. VR has been proven to provide fairer results with age, so assessments applied at different ages showed variations on the classical exams, but not on the memory VR exam [42]. VR has also been used to evaluate complex cognitive functions such as prospective memory, in the case of [43] authors confirmed the deterioration of this memory in the early stages of Alzheimer’s disease. In this way, VR has been inserted as a memory diagnosis and rehabilitation tool, but there are few IVR studies aimed at evaluating memory, which motivates the development of applications with different levels of immersion [44].

The intelligence that characterizes the human being requires normal functioning of cognitive abilities. For this reason, memory requires being part of exercise and rehabilitation processes when the situation requires it. Dietrichkeit et al. [45] present a study carried out in healthy people and others with psychosis, determining that the positive effects that VR generates on the memory of the second group are greater. Mitrovic et al. [46] and Krokos et al. [47] present proposals for immersive systems to promote the development of prospective memory, in daily and long-term tasks, respectively. In Ventura et al. [48], the performance of episodic memory is compared when using immersive and non-immersive systems in healthy people. In this way, it is determined that the participants in the first group can recall a greater number of long-term events compared to the second.

Some memory-oriented IVR applications have been tested in healthy and patients that have some cognitive impairments to determine their effect on participants. Corriveau et al. [49] used a virtual store to memorize 12 familiar items and the results indicated that there were high levels of presence and higher levels of motivation for the VR tool in comparison with the traditional task. They also pointed out that memory performance in the VR task was positively correlated with performance on a traditional memory task in different age groups. Bréchet et al. [50] analyzed the first-person influence on IVR for virtualized episodes of everyday life and determined that delayed recovery performance can be improved when participants view their body as part of the virtual scene. Ouellet et al. [51] presented a virtual store to evaluate the daily memory in participants with and without cognitive impairment, whose results were validated with traditional questionnaires.

Cognitive functions deteriorate with aging and neurodegeneration, as has already been seen in the described literature. For this reason, motivating and effective proposals should be implemented to train these functions from middle age, preventing the advent of long-term mental problems. The type of system proposed (immersive or non-immersive), age, and other inherent physical and biological factors of the participants must be taken into account, in order to offer an appropriate tool for each of them. Several investigations were developed using immersive environments focused on the different types of memory, with a greater preponderance to the long-term ones. In this context, this manuscript presents an interactive system using IVR for assessing and training memory. An application that includes virtual environments was developed in the Unity 3D video game engine and the Oculus Go (HMD) headset was used as an immersive device. The main contribution of the current work is the presentation of a low-cost solution intended for memory training purposes that can also be used in community-based environments.

This work is organized as follows. Section 2 includes the materials and methods, with the design of the proposed task to assess memory, its main features as well as the conducted experiments to validate the application. Section 3 presents the main results. Section 4 is devoted to the discussion and Section 5 deals with the conclusions and future work.

## 2. Materials and Methods

### 2.1. Formulation

A usable app designed to train memory using cognitive stimulation exercises is required. Traditional cognitive rehabilitation strategies consist of ordering patterns, memorizing events and performing mental operations, normally carried out in printed notebooks or using digital screens. There are also personalized cognitive stimulation sessions that require a specialist, which implies a greater investment of time, infrastructure, and money. These commonly applied tools can generate some type of stress to the user, reduce motivation and degrade treatment results.

**Figure 1:**
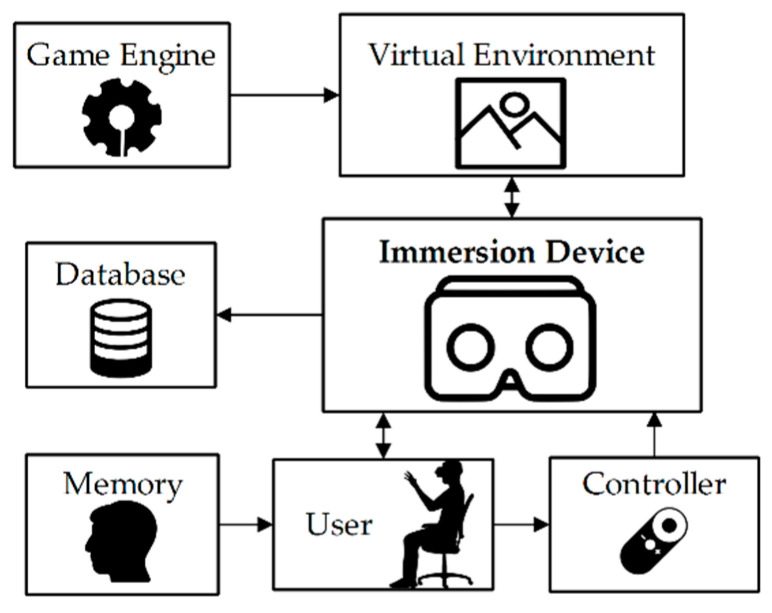
Formulation of the memory rehabilitation problem using an immersive virtual reality application.

The technology has an infinity of applications in rehabilitation. Specifically, VR combined with serious games has positive effects on the user, emulating therapeutic activities as entertainment. In addition, the use of immersion devices allows to improve concentration in cognitive rehabilitation, excluding distractions present in the real environment. Figure 1 presents the proposal described in this document, where VR is used for the patient to interact with a set of predefined cognitive exercises through a hand controller. The system provides a virtual environment developed in a game engine, where all the visual and auditory information is played on the immersion device. The results of the games are also saved in a database for later review and analysis.

The proposed application differs from traditional techniques since the rehabilitation strategy consists of memorizing patterns while carrying out other interaction activities with the virtual environment. In this way, greater cognitive activity occurs, and the actions are fed back with sound to reinforce the immersion in the environment. The game is designed so that the user makes minimal movements, considering that the player’s position must always be seated on a fixed chair to avoid collisions with objects in real space. In addition, the specialist can access the result history from anywhere, because the database sends the stored information to a file transfer server.

Within the serious game, the user has to walk through a maze in first person using the HMD device. While moving through the corridors geometric shapes appear, and the user has to remember both the color and the number of appearances. The analysis is carried out from two modes, autonomous and manual locomotion (user-controlled mode). In the autonomous mode, the player is automatically taken to the exit of the maze, whereas in the user-controlled mode, the player has to do it by commanding the Controller.

### 2.2. Components

Regarding the components required for this proposal, this focuses on implementing a wearable rehabilitation system that allows the user easily locate it anywhere in the home and it is economically accessible. Similarly, versatile and developer-friendly software (game engine) is required, which makes it easy to use immersion devices and enables the application to be linked to the file transfer server. In the current market, there is a great variety of HMDs with different input devices, as well as multiple game development environments. Consequently, the mentioned aspects are considered to select the necessary elements.

**Figure 2:**
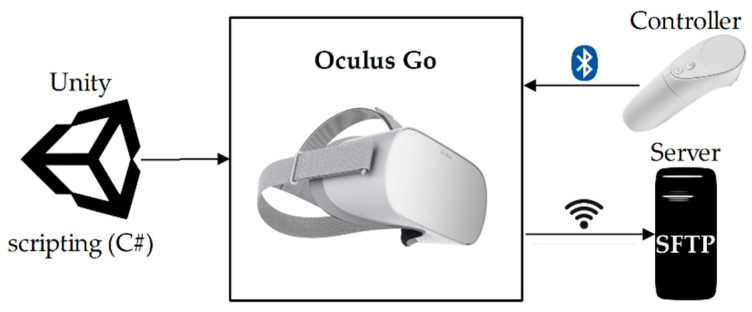
Components of the immersive virtual reality application.

Figure 2 shows the components selected to implement this proposal, the HMDs to use are the Oculus Go that belong to the Oculus VR Company belonging to Facebook. This VR device is the successor of the Gear VR and its main feature is independence, since it does not require wiring, nor a Smartphone, nor a computer for its operation. Oculus Go is an all-in-one VR viewer for its display and built-in audio. In connectivity, it has Bluetooth 4.1 technology to communicate a single wireless controller, and it has Wifi 802.11ac communication to connect to the network.

Technically, the HMD have the following characteristics: Android 7.1.2 operating system, system installed on a Qualcomm Snapdragon 821 chip, 3Gb LPDDR4 memory, 64 Gb storage (latest model), screen resolution of 1280 × 1440 per eye, frequency 60 to 72 Hz refresh rate, 101 degree field of view, density of 12.67 pixels per degree of vision, Adreno 530 graphics processing unit, dimensions 190 mm × 105 mm × 115 mm, mass 468 g, integrated battery 2600 mAh for a range of two hours of play, and a price of approximately US $ 200.

The remote input controller incorporated in the Oculus Go has an inertial measurement unit (IMU) to track 3 degrees of freedom of angular movement (useful as a pointer to interact with the virtual environment); it also includes a circular touch panel and four push buttons (Trigger, Back, Touchpad and Oculus). For its operation, a single AA battery powers it.

The Oculus Go have development compatibility with Unreal and Unity 3D, of these two game engines, the second has a more versatile and easy-to-develop work environment. Unity is a video game development platform with online support documentation, and has cross-platform compilation support. To build virtual environments Unity use the PhysX physics engine, animations using Mechanism technology and scripting is implemented in C# (.NET Framework). In relation to costs, Unity has two versions, a free personal and a professional one that requires payment.

For file transfer, an SFTP server from the University of Zaragoza is used, which allows the results to be sent in a secure manner. This is possible thanks to the connectivity of the device with the internet through a wireless network and the Microsoft Visual Studio libraries for file transfer protocols.

The advantages of this immersion device are remarkable, favoring the development of the application proposed in this work. The comfort of independence and the acceptable weight of the glasses are some of the benefits found. However, the excessive use of these HMD produces undesirable effects, some of the symptoms are dizziness, nausea, and vertigo, and worse still, when the user has health problems the risk increases, among the consequences are epileptic seizures, for this reason it is important to consult the player’s background before considering him a participant in this proposal.

### 2.3. IVR Game Design

There are several possibilities to design a tool that exercises memory, but the most important thing is to provide it with simplicity to facilitate the use of the application and the analysis of the results. The game was initially designed to memorize geometric shapes within the virtual environment so that as the player progresses, more elements to memorize appear. The basic geometric shapes and the quantity of each one must be memorized; additionally the color assigned to each group of figures must be remembered, according to the level of difficulty selected. The user’s journey is within a maze, from the entrance to the exit, solving the maze in first person. On the other hand, the maze navigation mode has two modes, the autonomous mode where the player only concentrates on memorizing the geometric shapes and their colors, and the manual mode (user-controlled mode) where the player controls the movements to find the exit. This last mode adds more elements to the cognitive exercise and allows analyzing the solution times of the maze.

**Figure 3:**
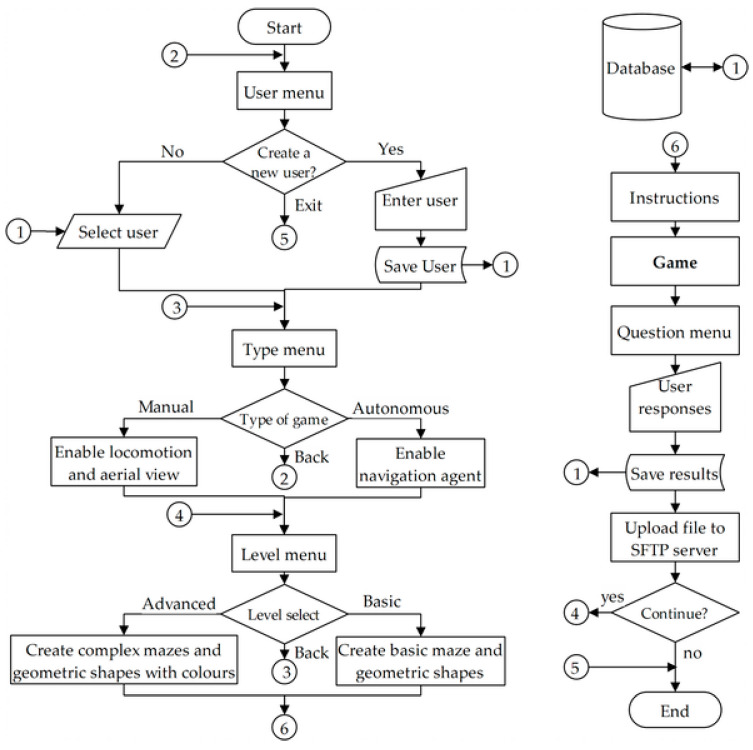
Algorithm of the cognitive rehabilitation application.

Figure 3 presents the entire algorithm of the application from start to finish. Initially the user menu is displayed to record the player’s data. This menu consists of an alphabetical keyboard to enter the user’s names, considering that it can be selected from a pre-existing list of players, where the execution data is read and stored in the database and additionally an option is included to exit the game at any time. Then, a menu is presented to decide the navigation mode within the maze. In the autonomous mode, the navigation agent is enabled, whereas in manual mode, controlled locomotion is enabled and reproduces an aerial view to identify the way to the exit of the maze. This menu has the option to return to the previous menu.

**Figure 4:**
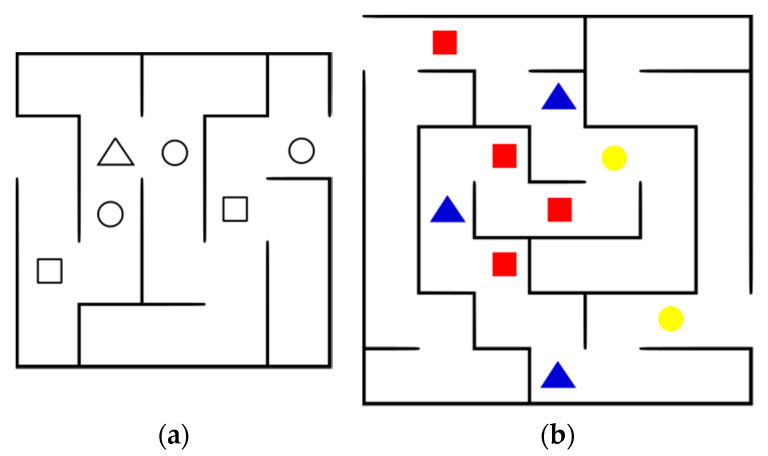
Difficulty levels: (**a**) Basic; (**b**) Advanced.

Below is the difficulty level menu, offering two options. The basic level (BL) generates a maze of low difficulty and locates the geometric shapes (square, triangle and circle) randomly (see Figure 4a). The advanced level (AL) generates a more complex maze and locates the geometric shapes with their respective colors (red, green and blue), one color for each type of shape (see Figure 4b). Regarding the generation of the maze, the internal configuration is produced in a random way so that the mazes are not repeated, in terms of the number of shapes, a maximum of ten are created for each shape, and the colors are also assigned randomly, this menu also has the option to return to the previous menu.

Returning to Figure 3. Once the level is selected, the instructions for the game are displayed for a certain time, the aerial view is played (in manual mode) and the game begins, considering that at any time the user can close from the game and return to the level menu. When the player reaches the exit of the maze, the menu of questions related to the number and color of the shapes is displayed. Entering the answers ends the game, saving the results in the database and sending the file to the SFTP server. Finally, the user decides whether to continue playing or exit the application.

### 2.4. Development of the VR Application

**Figure 5:**
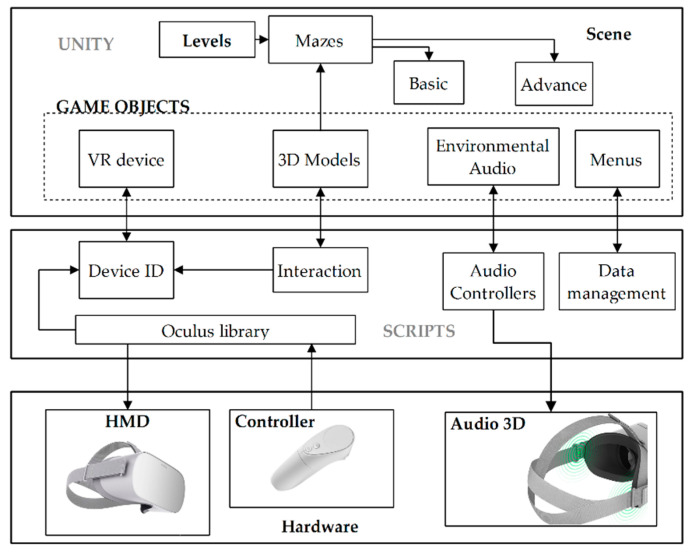
Diagram of the development of the immersive virtual reality application.

The application is developed using the Unity 3D engine of game. Figure 5 presents the implemented scheme to develop the game; the main components are Game Objects and Scripts. The Unity environment allows creating the game scene, where all the Game Objects are inserted, starting from the camera that is linked to the VR device, the 3D models that make up the virtual environment and the two levels of difficulty of the maze, the sources of audio, and the different interaction menus, each component has a functionality that is controlled from the Scripts.

Scripts contain C# programming to link the different actions of the application. To implement these programs, several libraries are used that allow access to the hardware and functions required by the system. The interaction between external devices and Game Objects is done in different scripts, as well as events for audio playback. Data handling requires specific libraries that allow you to create, edit, and store the files, which also use the network connection to send the files to the remote server.

**Figure 6:**
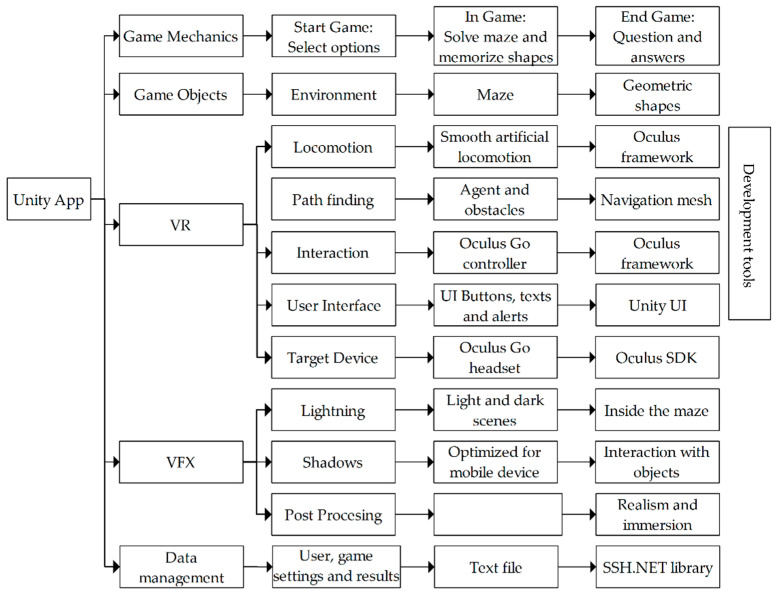
Developed elements of the virtual reality application.

Next, the elements developed for the VR application are described, as shown in Figure 6. In the game mechanics, the links are made between all the menus from the start of the game to the user’s responses. The program activates and deactivates the Canvas as required, the menus are mainly made up of buttons, but it also uses dropdowns to show the options of existing users and the responses. In the game, invisible colliders are created to detect when the user enters and exits the maze, which allows measuring time and activating the menu of questions. There are also colliders in the geometric shapes that are continuously rotating. They disappear on contact with the player and give a feedback to the user by means of a sound.

The visual Game Objects are those that make up the scene environment, the maze and the geometric shapes. The environment consists of a field with grass, small country houses, water, trees, and stones, these elements were imported from different Assets. Mazes are generated automatically using a Pure Recursive cell-based algorithm, this 3D model is made up of three Prefabs (Walls, Pillars and Floors) that are located in the positions generated by the algorithm. The geometric shapes are positioned in the maze spaces at random and are Prefab with animation to rotate continuously.

Player movements are run using the Smooth Artificial Locomotion technique; the Oculus framework allows reading data from the Controller’s touchpad using the OVRInput.Get function (OVRInput.Axis2D.PrimaryTouchpad), to affect the position and orientation of the camera (kinematic) using the Transform property.

Autonomous navigation is possible thanks to the Path Finder of Unity 3D that allows you to navigate from one point to another automatically and avoiding obstacles. For this reason, it is necessary to add a NavMeshAgent to the player and an additional NavMeshObstacle to the possible collision objects, where a flat is created and set as Navegation Static to generate a Bake where the player can move. To activate this mode in the Script, the destination is configured and the Agent.isStopped is turned off. On the other hand, aerial view (manual mode) requires disabling the player’s navigation agent to raise the camera.

The user interaction with the menus is done through a virtual pointer linked to the movements of the Oculus Go Controller, this works through a Line Renderer that detects the collision with the elements of the Canvas, to confirm the selection, press the Trigger button (OVRInput.GetDown (OVRInput.Button.Trigger)). The Back button allows you to stop a game and the Oculus button can be used to exit the application. Menus were created with Unity’s user interface (UI) tools and adding Colliders to buttons and dropdowns. To connect the virtual environment camera to the HMDs, the Prefab of the Oculus library called OVRCameraRig is used.

To conclude the edition of the virtual environment, the visual effects (VFX) are configured that produce a pleasant environment for the user. The lighting is adapted to distinguish some areas lighter than others, especially for the shadows generated within the maze. These shadows are generated using Lightmapping to optimize the consumption of resources on the mobile device, which improves interaction with objects. A post processing technique is also applied to improve the appearance on the camera and insert realism to the virtual environment; anti-aliasing softens the appearance of the graphics.

Finally, data management is implemented, where the information acquired is the username, the game configuration (type of game and level of difficulty), the correct answers, and the user’s answers. The data is stored cumulatively in text files and sent to the SFTP server through the SSH: NET library, using functions of the SshNet.Sftp type.

### 2.5. Activities and Participants

**Figure 7:**
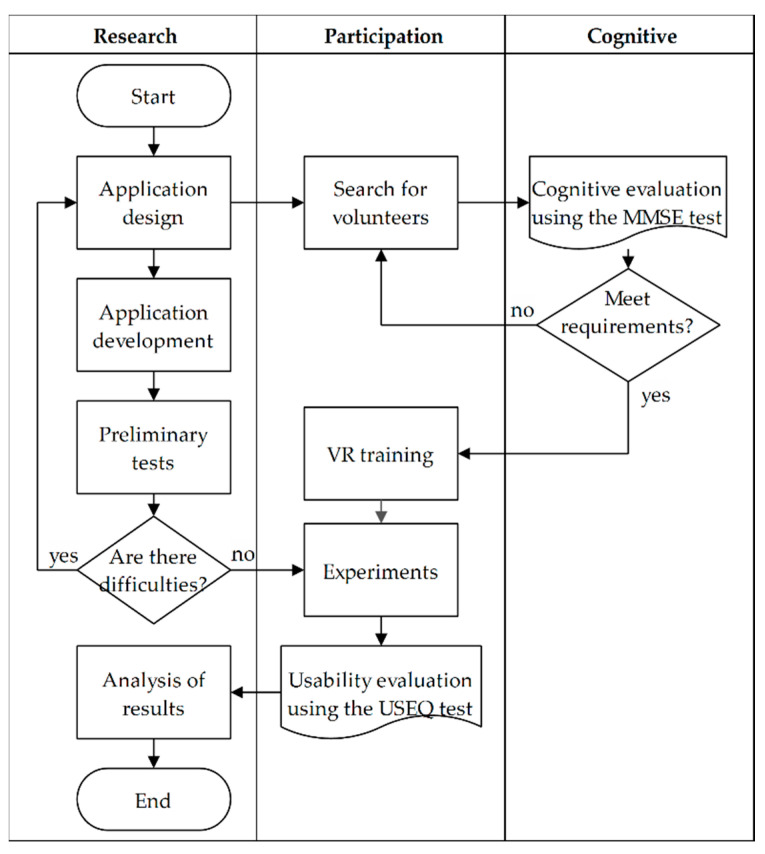
Process diagram of the activities carried out.

This proposal is implemented following the processes in Figure 7, based on the design of the application detailed in Section 2.3, then the VR application is developed according to Section 2.4. The preliminary tests of the serious game are then carried out, analyzing the characteristics of the virtual environment and revealing possible errors; any detected difficulty requires improvements in the application.

Several changes were made according to this previous evaluation:

- The text was not very legible at certain distances due to the small size, so text font in menus and game instructions was enlarged.
- The menus were located below the level of the horizontal line of sight, requiring tilting the head to see all the option, so the menus were located in a higher position.
- The textures on the walls of the maze had a kind of discordance that affected aesthetics. Textures were configured to eliminate such errors.
- The aerial view changed very quickly and did not present a good angle of vision. For that reason, time was corrected and a better position for aerial view was established.
- The speed (both linear and angular) of locomotion (both autonomous and manual modes) were producing dizziness in the user; speed was reduced for the sake of comfortless.

Parallel to the development of the VR application, activities related to the participants are carried out, starting with the search for volunteers. The minimum requirements to consider a volunteer are to be at least 16 years old and agree to collaborate with the research, where the interested parties approved the informed consent to carry out the evaluations. Not all volunteers performed the experiments with the VR application, before a cognitive evaluation was performed using the Mini Mental State Examination (MMSE) test with adaptations to the participants [52]. The objective of this exam is to detect a possible deterioration in brain function that affects the use of the application, consequently, the results of the experiments, since this proposal is evaluated in healthy subjects of different ages.

Table 1 contains the MMSE test questions with their respective scores (total 30 points), this evaluation consists of 11 items. Temporal orientation -1- is analyzed by asking the volunteer for five data related to time, and spatial orientation -2- with five geographic location data. In the memory fixation -3- the patient is asked to repeat three words. In the attention or calculation -4- item, 3 is subtracted from the number 20 by 5 times, in case of presenting problems, it is asked to say the days of the week upside down. The memory -5- is evaluated by asking the words mentioned in the memory fixation. The denomination -6- is analyzed with the description of two objects. Then the user is asked to repeat -7- a phrase, and the language -8- is evaluated by reading and doing what a text indicates. Order tracking -9- is reviewed using three instructions given to the user, then the patient must write -10- a complete sentence (subject and predicate), and finally, the ability to copy -11- is evaluated by replicating two crossed pentagons using sticks.

The inclusion criteria to select the participants were: an MMSE score greater than 26, not having excess sensitivity to movement or motion sickness, and having no brain disorders or lesions that produce epilepsy. In total, 29 participants were recruited from the city of Ambato (Ecuador), whose characteristics are presented in Table 2. As far as formal education is concerned, participants had an average of 15.28 years (SD = 4.5), 62% had university studies and 38% did not. Overall, 52% were male and 48% female, 41% had no experience with VR and only 17% exhibited full experience, 55% carried out cognitive exercise regularly (for their own interest or because they worked in cognitive activities), and only 14% had a family history of neurodegenerative diseases. The average age of the participants was 31.41 (SD = 10.6) years old (ranged 16 to 64), and the group’s average score on the MMSE test was 29.07/30.

In order to contrast the results obtained in our maze task, a control test was carried out by participants. The control test was performed using a face–name pairs test. This test was similar to the common memory tests about face–name pairs we can find in the literature [53,54]. In this case, we used a short-time version that did not require several days for acquisition like in [53]. In the experiment, each participant had to memorize a sequence of 30 named faces (using 6 s per image), and then had to match faces with their respective names. We stored the response accuracy and completion times for every participant.

Prior to the experiments, all participants were trained in virtual reality using the Oculus Go, this to level the expertise in the use of IVR. Then, in the experiments, the participants generated results periodically and under similar conditions. After finishing the experiments, participants answer a simplified 6-question usability test to evaluate the VR application.

### 2.6. Statistical Analysis

We conducted Pearson’s correlation tests to determine whether there were a positive and significant correlation between the participants’ response accuracy in both the face–name pairs test and our maze task. We also evaluated the correlation to determine whether the years of formal education showed a significant positive correlation with performance on the task and to determine if the response accuracy or the completion times were sensitive to the changes related to age. T-student test were conducted when examining the effect of education on accuracy as well as to find for differences by gender in both response accuracy and completion times. The statistical significance was set at *p* < 0.05.

## 3. Results

### 3.1. Virtual Environment

**Figure 8:**
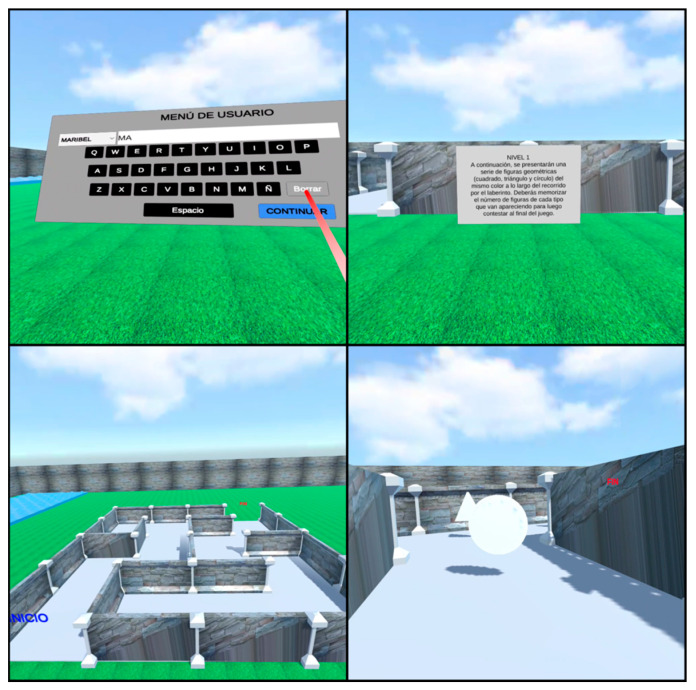
Immersive virtual reality scene views at the basic level.

After implementing the final corrections to the application, the final VR environment is obtained. Figure 8 presents some views of the basic level, showing the user menu with the keyboard to enter the names, the pre-game instructions, the maze from the aerial view, and the interior of the maze with the colorless geometric shapes. All the information in the system is presented in the Spanish language because the participants are Spanish speakers.

**Figure 9:**
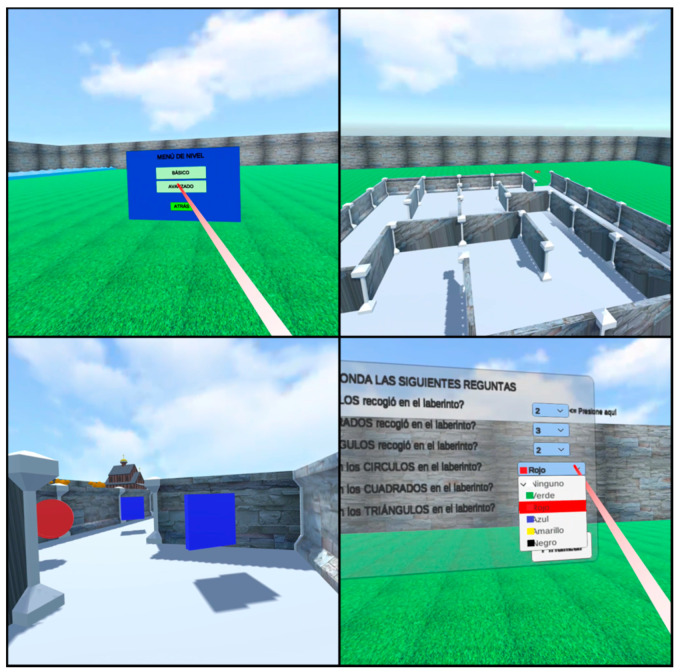
Immersive virtual reality scene views at the advanced level.

Figure 9 shows more images of the virtual environment, this time the level menu, the maze of the second difficulty level, the interior view of the maze with colored geometric shapes, and the question and answer Canvas for the end of the game are presented. The landscapes are visibly attractive and the programmed functions work correctly. Due to the optimization of resources, the application requires a space of 45 Mb for its installation.

### 3.2. Experimentation

**Figure 10:**
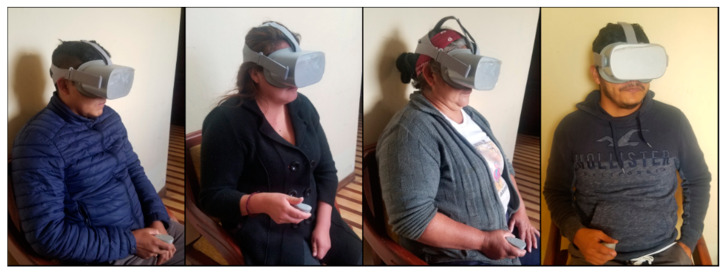
Participants using the immersive virtual reality application.

The tests were carried out with the participants by means of two Oculus Go VR Headset working simultaneously. Participants initially were trained for two weeks to become familiar with the equipment. Figure 10 shows some participants using the application, the experiments were carried out for 1 month and each participant performs at least ten tests in each of the game levels (basic and advanced).

#### 3.2.1. Autonomous Mode

**Figure 11:**
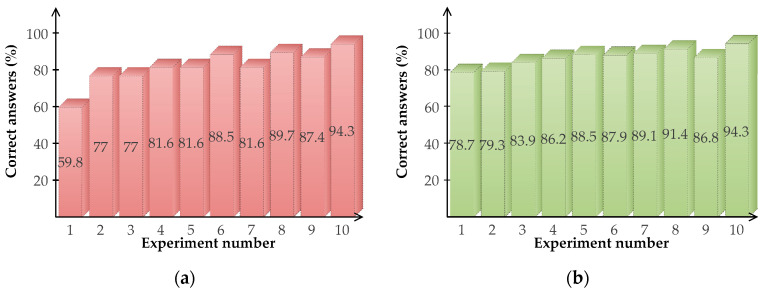
Results of the immersive virtual reality application in the autonomous game mode: (**a**) Success at the basic level; (**b**) Success at the advanced level.

Figure 11 depicts the results of the participants in the autonomous mode, where the players only memorize the geometric shapes without worrying about the movements within the maze. At the basic level, there is a maximum of three correct answers per test, and the participants performed ten tests. At the advanced level, there is a maximum of six correct answers per test and participants performed ten tests to accomplish the autonomous mode task. Across all the participants, mean response accuracy (correct answers percentage) was high (85% on average), being higher for the advanced level (86.6% on average) regarding the basic one (81.8% on average). All participants achieved accuracy at or above 50%. The total time to accomplish the task was 65 s for the first level and 97 s for the second one. Figure 11 shows the results of the experiment for both levels basic (a) and advanced (b).

Significant differences were found in the mean response accuracy by gender in the whole of the task (t (27), *p* = 0.046) although not in each one of the levels of difficulty (BL: t (27), *p* = 0.065, AL: t (27), *p* = 0.278). In this case, males performed better than females (M = 87.7, SD = 6.06% versus M = 81.7, SD = 9.3).

**Figure 12:**
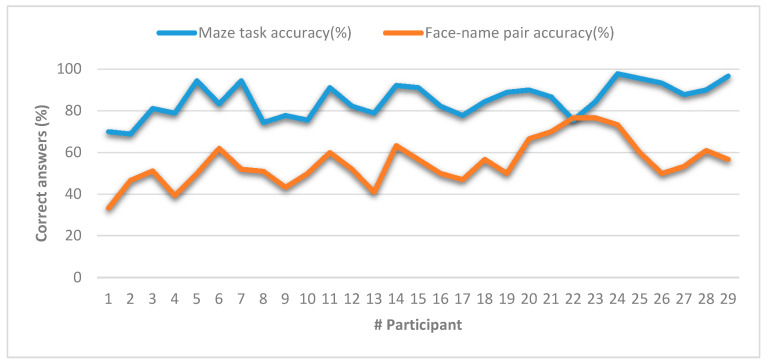
Comparison of memory measurements between the maze task and the face–name pair test in autonomous mode.

When examining the effect of education on accuracy, participants with a formal education greater than or equal to 14 years had a significantly better performance (M = 87.25%, SD = 7.43% versus M = 80.77%, SD = 8.01%; t (27) = 2.17; *p* = 0.039). In addition, the years of formal education show a significant positive correlation with performance on the task (r = 0.465, *p* = 0.011). In order to determine if the response accuracy in this task was sensitive to changes related to age, we evaluated the correlations between age with the accuracy of the response and we did not found any association. We also conducted the face–name pair test [54], broadly used for memory assessment purposes. Figure 12 compares the mean response accuracy for both the maze task and the face–name pairs test, where one can observe that both measurements methods followed similar trends and shapes. In order to corroborate such assumption, we conducted Pearson’s correlation test between the results of both memory measurement methods. Table 3 shows the results of this correlation test. According to the results, we found a positive and significant correlation between the participants’ response accuracy in both the face–name pairs test and our maze task (r = 0.435, *p* = 0.018), which was medium–high according to Cohen criteria [55].

#### 3.2.2. User-Controlled Mode

Within this mode, players memorize geometric shapes and control the movements within the maze. The number of performed tests by participants was the same as in the autonomous mode (ten tests in each level).

There were no significant differences in the average response accuracy by sex neither in the task as a whole ((t (27), *p* = 0.195) nor within levels of difficulty (BL: t (27), *p* = 0.193, AL: t (27), *p* = 0.405). The same applies in terms of global ((t (27), *p* = 0.934)) and partial (BL: t (27), *p* = 0.704, AL: t (27), *p* = 0.869) completion times.

**Figure 13:**
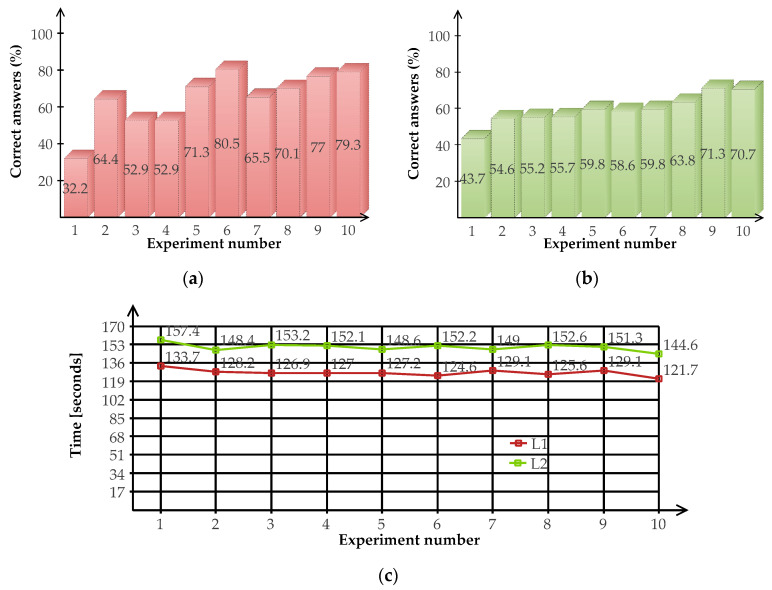
Results of the immersive virtual reality application in the user-controlled mode of the game: (**a**) Success rate at the basic level; (**b**) Success rate at the advanced level; (**c**) Maze completion times.

Across all the participants, the average response accuracy (percentage of correct answers) was moderate (61% on average), being higher at the basic level (64.6 %) in comparison with the advanced level (59.3%). All participants except one (46.67%) exhibited an accuracy greater than 50%. The total completion time of participants as a whole ranged between 105.15 and 155.04 s. The average total completion time was 139.35 (SD = 10.43) seconds. The average completion times for the basic level and advanced level were 127 and 152 s, respectively. Figure 13 shows the participants’ results in the user-controlled mode (both response accuracy and completion times, respectively).

By examining the effect of education on response accuracy and completion time, participants with a formal education of 14 years or higher performed significantly better (M = 63.68%, SD = 8.4% versus M = 56.11%, SD = 4.54%; t (27) = 2.63; *p* = 0.014). Furthermore, the years of formal education show a significant positive correlation with the performance on the task (r = 0.463, *p* = 0.01). We did not find any association with the total completion times of the task.

**Figure 14:**
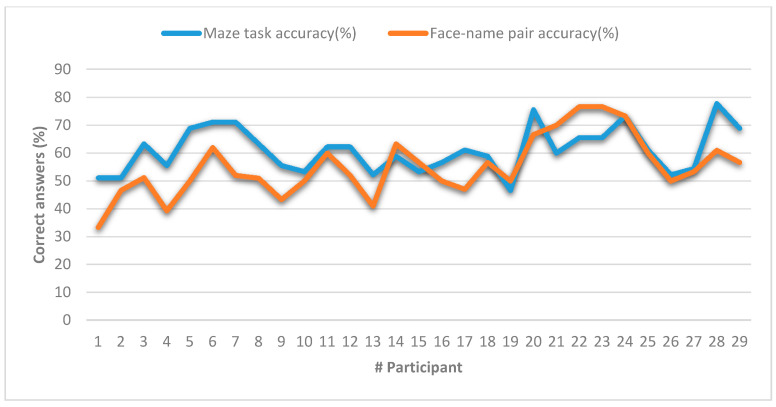
Comparison of memory measurements between the maze task and the face–name pair test in user-controlled mode.

In the same way, as we did in the autonomous mode, we compared the mean response accuracy for both the maze task and the face–name pairs test (see Figure 14). In order to confirm if a linear correlation may exist, we conducted Pearson’s correlation test between the results of both memory measurement methods. Table 4 shows the results of this correlation test. According to the results, we found a positive and significant correlation between the participants’ response accuracy in both the face–name pairs test and our maze task under a user-controlled mode (r = 0.577, *p* = 0.001), which is high according to Cohen criteria [55].

Finally, to determine if the response accuracy or the completion times in this task were sensitive to the changes related to age, we evaluated the correlations between age with the response accuracy and with the completion time for the different levels of the task (BL and AL). Age was not significantly associated neither with response accuracy nor with the completion time in the two levels of the task (*p* > 0.05 in both cases). The same happened (no significant association for completion times and response accuracy in the face–name pair test).

### 3.3. User Evaluation

To evaluate the application from the perspective of the participants, the USEQ satisfaction test (User Satisfaction Evaluation Questionnaire) [56] was applied. It allows us to measure the usability of VR applications for rehabilitation and consists of six questions evaluated from 1 to 5. Table 5 presents the test results, obtaining an average score of 28.36 (SD = 0.46), showing a high degree of satisfaction of the participants.

In general, users express their satisfaction with this experience, mentioning that all the elements of the application are comfortable to use and the information presented is quite clear. At the beginning of the experiment, there were some participants dissatisfied with the application because they experienced dizziness at first, but then they adapted to the environment. In general, the participants think that the system can be helpful for rehabilitation purposes.

## 4. Discussion

VR has been explored as a cognitive rehabilitation tool [37,38,39,40]. Specifically, IVR has not reached its maximum expression in rehabilitation due to the side effects it produces [39,40], although there are results that show a better response in immersive systems for cognitive rehabilitation [46,47,48,50,51]. We believed that IVR systems based on a head-mounted display (HMD) offer many advantages in comparison with traditional treatments. Namely, customization based on user’s needs, possibility to graduate, low cost, more in terms of presence and ecological validity [57].

The main goal was to develop a task to assess the memory by exploiting the benefits of an IVR environment. At the same time, the task was intended for memory training purposes, especially focused on attentional memory. The application was developed using basic elements of the Unity 3D game engine, which has optimized the features and reduced the memory space required. Furthermore, the system constantly collects the generated information and sends it to an external server that allows the results to be easily extracted. The results based on by 29 patients of different ages (ranged 16 to 64) showed that the accuracy of participants measured in percentage of correct answers in a controlled environment was correlated (statistically significantly) with the response accuracy of participants in retrieving face–name associations in a validated type of memory test.

The observed correlations between the response accuracy in both the maze task and the face–pair test [54] align with other studies. In particular, González-Landero et al. [53] designed a smart cupboard (SC) as a tool to perform memory measurements and they also found a significant correlation between the accuracy response in both the SC and face–pair test for a sample of 23 adults. Like in their study, we observed that the participants’ results of the face–pair test were well below the results achieved by the participants in the maze task. One plausible explanation deals with the fact that attention appears to be able to influence items already stored in visual memory. Our application works sustained attention, since it works the ability to focus and maintain attention on a task over a long period [58,59]. This idea is in line with the study of Murray and colleagues [60], who showed that selective attention could restore forgotten items to visual short-term memory (VSTM). Therefore, if we direct attention to items already encoded in memory, the probability of their recall improves. Furthermore, they stated that attention could rescue information that would otherwise be lost or unavailable to retrieval processes. Other authors such as Zang and Luck, demonstrated that attention protects items from being forgotten [61], whereas Matsukura and colleagues’ work suggested that attention can assist in selectively encoding items into visual memory [62]. This suggests to us that perhaps we should bring these values closer together, probably by increasing the difficulty of the task. In fact, in the user-controlled mode (more difficult for the user) the correlations between our task and the face pair test were greater. It opens the door to new designs in which the difficulty of the task is slightly increased.

On the other hand, the results of completion times in the maze task indicated that there were important differences between the two modes (autonomous and controlled). The user-controlled mode involves introducing a distracting variable as an interference that made the completion more difficult for the participants (at both levels). In line with the abovementioned fact, the inclusion of some distracting elements can increase the difficulty and consequently, make the task more similar to realistic daily conditions.

Finally, in spite of the fact that some studies have reported side effects such as nausea or dizziness that are indicative of “simulator sickness” related to vestibular–optical reflexes [63,64], the participants did not present considerable discomfort with the application because the player’s speeds were adjusted to a minimum to avoid dizziness and other side effects of the headset. This was achieved despite the wide age range of the users who evaluated this proposal, although some users wanted to increase this speed, at the risk of having these effects, which is not convenient if we want to use the application on patients in the future.

However, this study had several limitations. First, we did not compare non-treated controls, so it is difficult to evaluate the degree of effectiveness of our cognitive task. Instead of comparing it with a non-treated control, we compared the experimental group performance with a well-known test [54] by means of Pearson’s correlation.

We also find that neither the response accuracy nor the completion times in this task were sensitive to the changes related to age. As far as completion times and response accuracy in the face–name pair test, we also find any association, partly due to the low average age in the sample. This fact is very important because there is a deterioration of memory with age and in some neurodegenerative diseases. Therefore, we should consider a greater sample for future studies, and if possible, with more presence of older subjects, in order to elucidate whether the task can be sensitive either in response accuracy or in completion times to age. This will allow us to extract interesting conclusions for application to a population with neurodegenerative diseases. To support this theory, longer studies should be conducted over time in a population of subjects suffering some neurodegenerative diseases such as Alzheimer’s or Parkinson’s diseases, and at different stages of the disease evolution. Such studies could confirm our hypotheses: first, to evaluate if the developed task could serve as a training task to improve short-term memory and second, to evaluate if the task could help to detect memory losses and eventually detect the disease at earlier stages. The inclusion of people more likely to develop a neurodegenerative disease due to genetic factors, for example, could be of capital importance to perform a better evaluation of our tool.

## 5. Conclusions and Future Work

In this preliminary study, we developed a virtual reality-based immersive application intended for both memory assessment and memory training purposes. A low-cost device (the Oculus Go HMD headset) was used as an immersive device. The results on response accuracy based on 29 participants showed that the accuracy of our task was correlated (statistically significantly) with the response accuracy of participants in retrieving face–name associations in the validated face–pair test assessment. Therefore, we ascertained that our task to assess memory converges with other extant and popular memory assessments such as the face–pair test assessment.

Future work deals with the creation of a robust tool for cognitive rehabilitation purposes by exploiting the benefits of immersive and low-cost technologies. They open the door to studies to elucidate which is better and under what conditions; taking into account the type of subjects/patients, age, etc. Thus, we intend to establish experiments with control groups that use a non-immersive technology compared to experimental groups using purely immersive technology through HMD such as the Oculus Go headset. We want to evaluate the tool in community-based environments before stepping into clinical settings. It is our goal to provide the tool with virtual environments closer to reality, and specifically close to activities of daily living (ADL), since the obtained results in the experimental setups are better transferred into daily life.

The obtained results also showed improvements in the memory retention of users as they use the application, especially when they are only concerned with memorizing geometric shapes (autonomous mode). This can lead us to use the tool for training purposes too [65]. The developed application also works attention, which is very important in diseases such as Alzheimer’s, where one of the possible neuropsychological markers deals with the attentional deterioration those patients may suffer in the preclinical phase of the disease. Thanks to mental dexterity tasks such as the one developed in this study, we can stimulate these cognitive functions. The application can also warn us of a deficit in sustained attention that may occur in other neuropsychiatric disorders. With this idea in mind, we plan to evaluate other cognitive functions such attention, immediate recognition memory, etc., in the same way as in the study of Kang [66].

Another line of this work is related to the introduction of cueing in cognitive tasks. Murray and colleagues showed that a specific type of cueing may improve recall for items that otherwise would have been irretrievable, providing critical evidence that attention can restore forgotten information to VSTM [60]. We think that immersive technology may improve that process.

We have left in the air one of the modes of operation, the user-controlled mode, in which the user must manage his journey through the maze to find the exit and finish the test. We intend to work on it in the future as well, to investigate how or to what extent interference that occupies our working memory capacity affects final performance. We hypothesize that adequate and sustained training over time can improve our working memory, and, consequently our short-term memory [67].

## Figures and Tables

**Table 1:** Mini mental state examination (MMSE) with adaptations.

Item	MMSE (Adaptation)	Score
1. Orientation in time	Year, date, Month, day, time of day	/5
2. Orientation in space	Place, floor, city, region, country	/5
3. Memory fixation	Apple, car, cow	/3
4. Attention—Calculation	Subtract 3 from 20 up to 5 times, or quote the 7 days of the week but upside down	/5
5. Reminder	Three objects learned instead	/3
6. Denomination	Pencil, shoe	/2
7. Repetition	“no, no, no, and no”	/1
8. Language	Read and do what is written: “close your eyes”	/1
9. Orders	Each individual is asked to follow the orders: ‘take this leaf, fold it in half, throw it on the ground‘	/3
10. Writing	Each individual is asked to write a sentence	/1
11. Copy	Each individual is asked to draw the figure of two pentagons using 10 sticks	/1

**Table 2:** Characteristics of the participants

Demographics	Data	Demographics	Data
Gender		Education	
Male	15	University	18
Female	14	Non-university	11
Age		MMSE	
Mean	31	Mean	29.07
SD	10	SD	0.87
Experience VR		Family history	
None	12	Yes	4
Some	12	No	25
Full	5	Cognitive exercise	
		Yes	16
		No	13

**Table 3:** Correlation between the response accuracy of both the maze task and the face–name pair test (autonomous mode).

		Maze Task	Face–Name Pair Test
Maze Task	Pearson Correlation	1	0.435 *
	Sig. (2-tailed)		0.018
	N	29	29
Face–Name Pair Test	Pearson Correlation	0.435 *	1
	Sig. (2-tailed)	0.018	
	N	29	29

* Correlation is significant at the 0.05 level (2-tailed).

**Table 4:** Correlation between the response accuracy of both the maze task and the face–name pair test (user-controlled mode).

		Maze Task	Face–Name Pair Test
Maze Task	Pearson Correlation	1	0.577 **
	Sig. (2-tailed)		0.001
	N	29	29
Face–name pair Test	Pearson Correlation	0.577 **	1
	Sig. (2-tailed)	0.001	
	N	29	29

** Correlation is significant at the 0.01 level (2-tailed).

**Table 5:** USEQ test results for the maze task.

Question	Mean	SD
Q1. Did you enjoy your experience with the system?	4.91	0.26
Q2. Were you successful using the system?	4.79	0.41
Q3. Were you able to control the system?	4.52	0.62
Q4. Is the information provided by the system clear?	4.97	0.18
Q5. Did you feel discomfort during your experience with the system?	4.45	0.85
Q6. Do you think that this system will be helpful for rehabilitation?	4.72	0.45
	28.36	0.46
